# Astaxanthin Inhibits Acetaldehyde-Induced Cytotoxicity in SH-SY5Y Cells by Modulating Akt/CREB and p38MAPK/ERK Signaling Pathways

**DOI:** 10.3390/md14030056

**Published:** 2016-03-10

**Authors:** Tingting Yan, Yan Zhao, Xia Zhang, Xiaotong Lin

**Affiliations:** alovelong@aliyun.comzx080750106@163.com18766312033@163.com

**Keywords:** astaxanthin, acetaldehyde, apoptosis, oxidative stress, Akt, MAPK

## Abstract

Excessive alcohol consumption can lead to brain tissue damage and cognitive dysfunction. Acetaldehyde, the most toxic metabolite of ethanol, mediates the brain tissue damage and cognitive dysfunction induced by chronic excessive alcohol consumption. In this study, the effect of astaxanthin, a marine bioactive compound, on acetaldehyde-induced cytotoxicity was investigated in SH-SY5Y cells. It was found that astaxanthin protected cells from apoptosis by ameliorating the effect of acetaldehyde on the expression of Bcl-2 family proteins, preventing the reduction of anti-apoptotic protein Bcl-2 and the increase of pro-apoptotic protein Bak induced by acetaldehyde. Further analyses showed that astaxanthin treatment inhibited acetaldehyde-induced reduction of the levels of activated Akt and cyclic AMP-responsive element binding protein (CREB). Astaxanthin treatment also prevented acetaldehyde-induced increase of the level of activated p38 mitogen-activated protein kinase (MAPK) and decrease of the level of activated extracellular signal-regulated kinases (ERKs). Activation of Akt/CREB pathway promotes cell survival and is involved in the upregulation of Bcl-2 gene. P38MAPK plays a critical role in apoptotic events while ERKs mediates the inhibition of apoptosis. Thus, astaxanthin may inhibit acetaldehyde-induced apoptosis through promoting the activation of Akt/CREB and ERKs and blocking the activation of p38MAPK. In addition, astaxanthin treatment suppressed the oxidative stress induced by acetaldehyde and restored the antioxidative capacity of SH-SY5Y cells. Therefore, astaxanthin may protect cells against acetaldehyde-induced cytotoxicity through maintaining redox balance and modulating apoptotic and survival signals. The results suggest that astaxanthin treatment may be beneficial for preventing neurotoxicity associated with acetaldehyde and excessive alcohol consumption.

## 1. Introduction

Chronic overuse of alcohol can lead to brain tissue damage and cognitive dysfunction [1]. Studies have shown that heavy drinking is associated with increased risk and earlier onset of dementia such as Alzheimer’s disease [2,3]. Ethanol is metabolized to acetaldehyde following consumption by alcohol dehydrogenase (ADH). In the brain, catalase and cytochrome p450 2E1 are additional enzymes that are critical for the local formation of acetaldehyde [4]. Acetaldehyde is further oxidized to acetate by aldehyde dehydrogenase (ALDH) 2. The level of acetaldehyde in blood elevates upon overconsumption of alcohol. Individuals with defective ALDH2 have much higher blood acetaldehyde levels after drinking alcohol in comparison with normal individuals [5].

Evidence has shown that acetaldehyde is the most toxic metabolite of ethanol. In various cell types, acetaldehyde is found to induce cytotoxicity and apoptosis [6,7]. Studies using cell or animal models deficient in ALDH2 or overexpressing ADH have shown that the cell damages and tissue injuries induced by alcohol are mediated by its metabolite acetaldehyde [8,9]. In central nervous system, it has been demonstrated that accumulation of acetaldehyde mediates the brain tissue damage and the cognitive dysfunction induced by chronic excessive consumption of alcohol. Locally-generated acetaldehyde is shown to mediate the acute inhibition of long-term potentiation by ethanol in the CA1 region of rat hippocampal slices and may contribute to the synaptic dysfunction [10]. In rat embryos, acetaldehyde treatment induces marked cell death in several tissues including neuroepithelium, correlating to the malformations seen in fetal alcohol syndrome (FAS) [11]. Acetaldehyde treatment also causes decrease in cell viability in rat cerebellar neuron cultures [12]. It has been shown that the cytotoxic effects of acetaldehyde are attributed to its induction of oxidative stress, mitochondrial dysfunction and activation of apoptotic signals such as cytochrome *c* release and caspase activation [12,13,14].

Astaxanthin (Ast) is a carotenoid that occurs naturally in a wide variety of organisms such as microalgae, yeast, salmon, trout, shrimp and crayfish. Currently, Ast is primarily produced from the green algae *Haematococcus pluvialis* and the red yeast *Phaffia rhodozyma* [15]. Ast has strong antioxidant properties. Studies have shown that Ast can effectively scavenge oxygen free radicals and is able to reduce lipid peroxidation and oxidative stress and inhibit reactive oxygen species (ROS)-mediated cytoxicity in both cell and animal models [16,17,18,19]. The function of Ast has been associated with a number of health-promoting benefits, including immunomodulation, prevention and treatment of cardiovascular diseases and cancer [20,21,22]. In addition, it has been reported that Ast reduces ischemic brain injury by inhibiting ischemia-mediated oxidative stress, glutamate release and apoptosis in the brain tissue in animal models [23,24]. Ast treatment has also been shown to suppress 6-hydroxyldopamine and glutamate-induced apoptosis in neuronal cells via attenuating pro-apoptotic signaling pathways and activating the expression of antioxidative enzymes [25,26]. These studies clearly demonstrate that Ast has neuroprotective functions.

As mentioned above, local accumulation of acetaldehyde is speculated to mediate the neurotoxic effects and brain tissue damages induced by chronic excessive consumption of alcohol. However, there are few reports regarding whether Ast can inhibit acetaldehyde-induced cytotoxicity in neuronal cells. In this study, we investigated the effects of Ast on acetaldehyde-induced cytotoxicity in human neuroblastoma SH-SY5Y cells. It was found that astaxanthin inhibited acetaldehyde-induced loss of cell viability and apoptosis. Ast treatment ameliorated the effect of acetaldehyde on the expression of Bcl-2 family proteins, preventing the reduction of anti-apoptotic protein Bcl-2 and increase of pro-apoptotic protein Bak induced by acetaldehyde. Further analyses showed that astaxanthin treatment attenuated acetaldehyde-induced reduction of the levels of activated Akt and cyclic AMP-responsive element binding protein (CREB). Ast treatment also prevented acetaldehyde-induced increase of the level of activated p38 mitogen-activated protein kinase (MAPK) and decrease of the level of activated extracellular signal-regulated kinases (ERKs). In addition, astaxanthin treatment suppressed the oxidative stress induced by acetaldehyde and restored the antioxidative capacity in SH-SY5Y cells. Therefore, astaxanthin may protect cells against acetaldehyde-induced cytotoxicity via inhibition of apoptotic signaling, promotion of cell survival pathway and suppression of oxidative stress.

## 2. Results

### 2.1. Ast Reduces Acetaldehyde-Induced Cytotoxicity and Apoptosis

**Figure 1:**
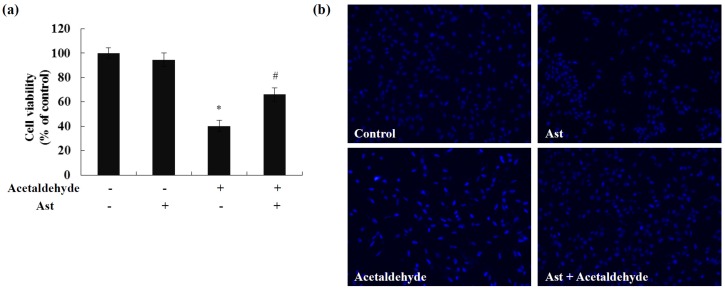
Effects of Ast on acetaldehyde-induced cytotoxicity and apoptois in SH-SY5Y cells. (**a**) Cells incubated with vehicle or 50 ng/mL Ast for 24 h were incubated with 10 mM acetaldehyde for 24 h. Cell viability was determined by trypan blue assay; (**b**) Cells were fixed and stained with Hoechst 33258. Values are means ± SD from three independent experiments. * Significantly different from control group (*p* < 0.05); # significantly different from acetaldehyde-treated group (*p* < 0.05).

It has been shown that acetaldehyde induces cytotoxicity in rat cerebellar primary neuronal cultures in a dose dependent manner [12]. Similarly, we have observed that acetaldehyde treatment decreases the cell viability of SH-SY5Y cells [27]. Here we tested the effects of Ast on the viability of SH-SY5Y cells stimulated with acetaldehyde. As shown in Figure 1a, after 24 h of acetaldehyde treatment, the cell viability was reduced to about 60% of the control cells. Ast pretreatment significantly inhibited acetaldehyde-induced loss of cell viability, leading to a 65% increase of viability in comparison to the cells treated with acetaldehyde only. Hoechst 33258 staining demonstrated that acetaldehyde induced apoptosis in SH-SY5Y cells while pre-incubation with Ast markedly decreased the number of apoptotic cells induced by acetaldehyde (Figure 1b). These results showed that AST treatment was able to prevent acetaldehyde-induced cytotoxicity and apoptosis in SH-SY5Y cells.

**Figure 2:**
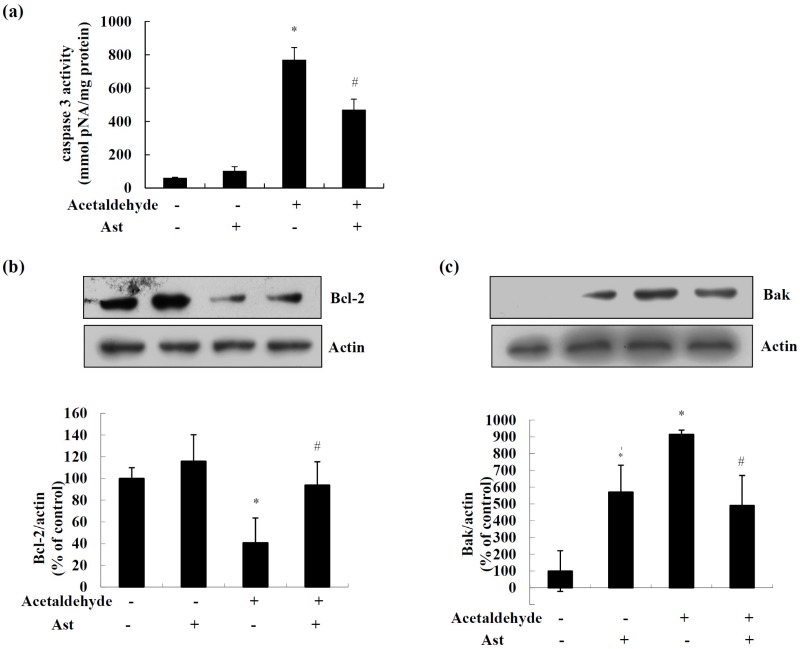
Effects of Ast on acetaldehyde-induced apoptotic signaling in SH-SY5Y cells. Cells incubated with vehicle or 50 ng/mL Ast for 24 h were treated with 10 mM acetaldehyde for 24 h. (**a**) Total lysates of cells were collected and the caspase 3 activities were determined; (**b**,**c**) Total cell lysates were collected and the amount of Bcl-2 and Bak was determined by Western blot analyses. The intensities of the bands were quantified by densitometric analyses and normalized by the amount of actin. Values are means ± SD from three independent experiments. * Significantly different from control group (*p* < 0.05); # significantly different from acetaldehyde-treated group (*p* < 0.05).

Caspase 3 is a hallmark of the late apoptotic events. As shown in Figure 2a, acetaldehyde significantly increased the activity of caspase 3 by nearly 12.5 folds, while Ast pretreatment inhibited acetaldehyde-induced activity of caspase 3 by nearly 40%. Mitochondria apoptosis pathway is mainly regulated by Bcl-2 family proteins via affecting the permeability of mitochondria membrane [28,29]. Thus, we next examined the effect of Ast on the expression of Bcl-2 family proteins. As shown in Figure 2b, the level of Bcl-2 protein was increased slightly but not significantly by Ast treatment alone. Surprisingly, we also observed that Ast treatment alone caused an increase in the level of Bak protein (Figure 2c). Exposure of SH-SY5Y cells to acetaldehyde for 24 h resulted in a decrease in Bcl-2 protein level by nearly 60%, while Ast pretreatment attenuated the loss of Bcl-2 protein (Figure 2b). Meanwhile, acetaldehyde exposure increased the protein level of Bak to nearly eight folds of the control cells, while Ast pretreatment markedly decreased this acetaldehyde-induced upregulation of Bak by approximately 50% (Figure 2c). These data suggested that AST might inhibit acetaldehyde-induced apoptosis of SH-SY5Y cells by modulating apoptotic signals including the expression of Bcl-2 family proteins and the activation of caspases.

### 2.2. AST Ameliorates Acetaldehyde-Induced Inhibition of Akt and CREB 

**Figure 3:**
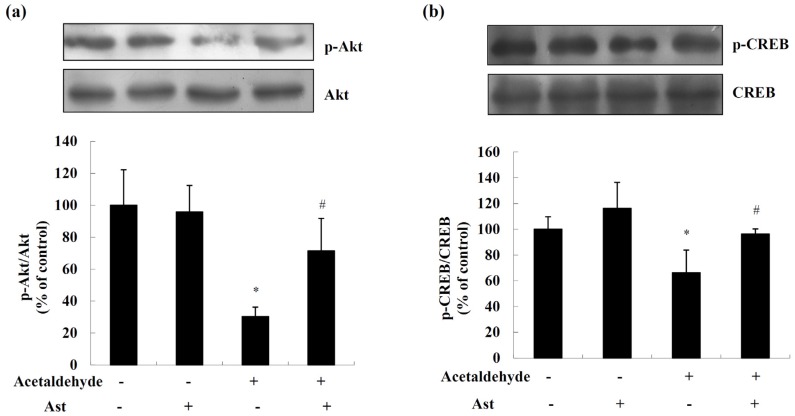
Ast treatment attenuates the inhibition of Akt and CREB induced by acetaldehyde in SH-SY5Y cells. Cells incubated with vehicle or 50 ng/mL Ast for 24 h were treated with acetaldehyde for 24 h. Total cell lysates were collected and the amount of Akt, phospho-Akt (Ser473), (**a**) CREB and phospho-CREB (Ser 133) (**b**) was determined by Western blot analyses. The intensities of the bands were quantified by densitometric analyses and normalized by the amount of Akt or CREB. Values are means ± SD from three independent experiments. * Significantly different from control group (*p* < 0.05); # significantly different from acetaldehyde-treated group (*p* < 0.05).

The phosphorylation and activation of Akt/CREB pathway are important for the survival of neuronal cells [30,31]. To examine whether Ast protects cells from acetaldehyde-induced cytotoxicity by promoting the Akt/CREB pathway, the levels of activated Akt and CREB were determined by Western blot analyses. As shown in Figure 3, acetaldehyde markedly decreased the levels of activated of Akt and CREB, by approximately 70% and 34%, respectively, while this reduction of activated Akt and CREB was significantly attenuated by Ast pretreatment (Figure 3a,b). These data suggested that Ast might inhibit acetaldehyde-induced cytotoxicity and apoptosis at least partly through preventing the decrease of the levels of activated Akt and CREB in cells treated with acetaldehyde.

### 2.3. Effects of AST on the Activation of MAPKs in Acetaldehyde-Treated Cells

**Figure 4:**
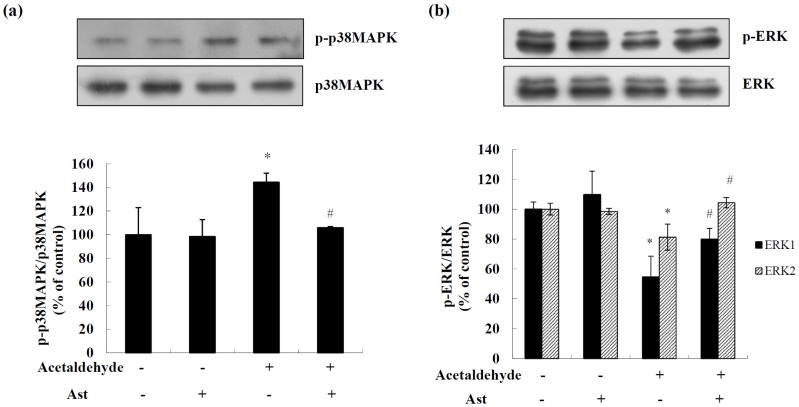
Effects of Ast on the activation of p38MAPK/ERKs in acetaldehyde-treated SH-SY5Y cells. Cells incubated with vehicle or Ast for 24 h were treated with acetaldehyde for another 24 h. Total cell lysates were collected and the protein levels of p38MAPK and phosho-p38MAPK (Thr180/Tyr182), (**a**) ERK (p44/p42) and phospho-ERK (Thr202/Tyr204); (**b**) were determined by Western blot analyses. The intensities of the bands were quantified by densitometric analyses and normalized by the amount of p38MAPK or ERK (p44/p42). Values are means ± SD from three independent experiments. * Significantly different from control group (*p* < 0.05); # significantly different from acetaldehyde-treated group (*p* < 0.05).

MAPKs have been shown to have important roles in promotion or inhibition of apoptosis. We next examined the effects of Ast on the activation of p38MAPK and ERK pathways in acetaldehyde-treated cells by Western blot analyses. Treatment of acetaldehyde dramatically increased the level of activated p38MAPK by approximately 44%, while Ast pretreatment for 24 h blocked this increase of activated p38MAPK, almost restoring it to the same level of that in control cells (Figure 4a). In contrast, acetaldehyde treatment caused a down regulation of the levels of activated ERK1 and ERK2 by approximately 46% and 19%, respectively, while Ast pretreatment significantly inhibited the increase of the level of activated ERK1 and ERK2, by 46% and 28%, respectively (Figure 4b). These data showed that Ast could block acetaldehyde-induced changes in the levels of activated p38MAPK and ERKs. 

### 2.4. Ast Reduces Acetaldehyde-Induced Oxidative Stress

**Figure 5:**
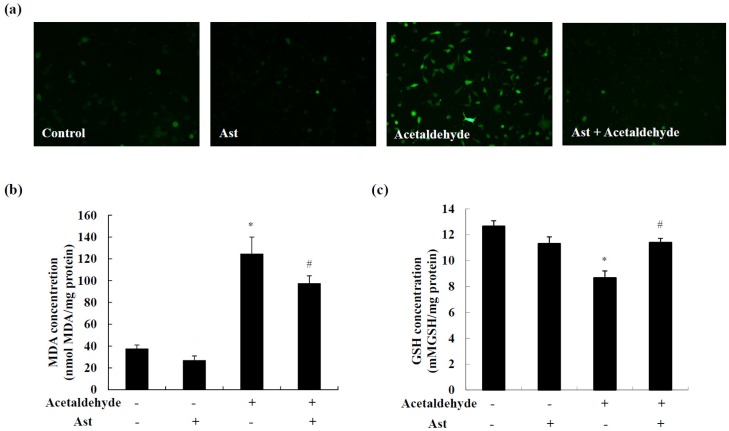
Effect of Ast on acetaldehyde-induced oxidative stress in SH-SY5Y cells. (**a**) Cells preincubated with vehicle or 50 ng/mL Ast were treated with 10 mM of acetaldehyde for 1 h. Cells were then stained with 2′,7′-dichlorodihydrofluorescin diacetate (DCFH-DA) to determine the production of ROS; (**b**) Cells preincubated with vehicle or 50 ng/mL Ast were treated with 10 mM acetaldehyde for 24 h. MDA production was measured in the cell lyates; (**c**) Cells preincubated with vehicle or 50 ng/mL Ast were treated with 5 mM of acetaldehyde for 2 h. The levels of reduced GSH were determined in the cell lyates. Values are means ± SD from three independent experiments. * Significantly different from control group (*p* < 0.05); # significantly different from acetaldehyde-treated group (*p* < 0.05).

Acetaldehyde has been shown to induce oxidative stress in neuronal cells [12]. Here we detected a quick and dramatic increase in the levels of ROS at 1h after exposure of acetaldehyde in SH-SY5Y cells. The induction of ROS production by acetaldehyde was greatly suppressed by the pretreatment of Ast (Figure 5a). Similarly, Ast also significantly reduced the level of malondialdehyde (MDA), a marker of lipid peroxidation and intracellular oxidative stress [32], in acetaldehyde treated SH-SY5Y cells (Figure 5b). Tripetide glutathione (GSH) is important for the detoxification of ROS and maintenance of redox status in the cell. It has been shown that GSH levels are significantly reduced at 2 h after acetaldehyde treatment in SH-SY5Y cells [27]. As shown in Figure 5c, Ast treatment prevented the decrease of the level of GSH induced by 5 mM acetaldehyde. The effect of astaxanthin on the levels of GSH was also tested in cells treated with 10 mM acetaldehyde; however, it was found that there was no significant effect of astaxanthin on acetaldehyde-induced reduction of GSH. As treatment with 5 mM and 10 mM acetaldehyde caused approximately 30% and 50% decrease in the levels of GSH, respectively, it was possible that the antioxidative capacity increased by astaxanthin might not be large enough to significantly prevent the reduction of GSH by 10 mM acetaldehyde. Nonetheless, these data suggested that Ast treatment attenuated the intracellular oxidative stress induced by acetaldehyde. As oxidative stress plays an important role in acetaldehyde-induced apoptosis and cytotoxicity, the antioxidant property of Ast may contribute greatly to the protective effect of Ast on acetaldehyde-induced cytotoxicity of SH-SY5Y cells.

## 3. Discussion

Acetaldehyde has been speculated to mediate the brain tissue damage and cognitive dysfunction induced by chronic alcohol overuse. Local accumulation of acetaldehyde in the brain can lead to neurotoxicity and perhaps contribute to the acceleration of the development of neurodegenerative disease. It has been shown that acetaldehyde induces neurotoxic effects by activating apoptotic signals such as downregulation of anti-apoptotic Bcl-2 family proteins and activation of caspases [12,13,33]. In this study, it was demonstrated that AST, a marine bioactive compound, protected neuronal cells from acetaldehyde induced apoptosis and cytotoxicity.

In response to apoptotic stimuli, pro-apoptotic Bcl-2 family proteins such as Bak are converted to oligomers that form large pores in the mitochondrial outer membrane, resulting in the release of apoptotic factors including cytochrome *c* into the cytoplasm and initiating a cascade of apoptotic events that lead to cell death [34]. In contrast, pro-survival Bcl-2 family protein such as Bcl-2 function in preventing the oligomerization and promoting the cytosol re-translocation of pro-apoptotic Bcl-2 family proteins, thus stabilizing mitochondrial membrane and preserving mitochondrial integrity, leading to the inhibition of apoptotic events [29]. Here it was shown that Ast ameliorated the effect of acetaldehyde on levels of Bcl-2 family proteins, inhibiting the reduction of Bcl-2 and increase of Bak induced by acetaldehyde. Thus, Ast might protect the cells from acetaldehyde-induced apoptosis by modulating the levels of pro-survival and pro-apoptotic Bcl-2 family proteins. In addition, it was found that Ast significantly decreased the acetaldehyde-stimulated caspase 3 activities. As Bcl-2 family proteins play key roles in regulating the expression and activation of caspase 3 [35], the decrease of caspase-3 activities by AST might be a result from its prevention of the downregulation of pro-survival Bcl-2 family proteins.

To our surprise, we found that astaxanthin treatment alone increased the level of Bak protein. It has been documented that Ast has anti-tumor activity [20]. In human and rat hepatocellular carcinoma cells, Ast has been shown to induce apoptosis while down-regulating anti-apoptotic Bcl-2 protein [36,37]. The concentrations of Ast used in these studies were at least 100 times higher than that used in our study, suggesting that Ast may promote cell survival at lower concentration but induce apoptosis at higher concentration. It is also possible that the effect of Ast on cell survival and apoptosis is cell specific, thus causing the contradictory results seen in different studies. No direct evidence has been shown that Ast modulates the expression of *bak* gene. It has been reported that p53 is involved in the induction of *bak* [38]. Recently, it has been shown that Ast increases the expression of p53 while blocking the oxidative stress-induced apoptosis in alveolar epithelial cells [39]. Therefore, it would be interesting to know whether p53 is elevated in Ast-treated SH-SY5Y cells and whether Ast modulates the expression of Bak at transcriptional level.

The serine/threonine kinase Akt is a key player in regulating cell signals that are important for cell death and survival [30]. It has been shown that active Akt protects SH-SY5Y cells against apoptosis through maintaining mitochondrial membrane potential and preventing the release of cytochrome *c* from mitochondria and the activation of caspases [40]. In this study, Ast treatment blocked acetaldehyde-induced reduction of activated Akt levels in SH-SY5Y cells. Transcription factor CREB is a downstream substrate of Akt. The phosphorylation of CREB by Akt on Ser133 results in its transcriptional activation and inhibition of apoptosis [31,41]. Consistently, we observed that decrease of the levels of activated CREB was attenuated by Ast treatment. CREB promotes cell survival via a transcription-dependent mechanism, upregulating the expression of anti-apoptotic genes such as Bcl-2 [42,43]. Thus, Ast may inhibit acetaldehyde-induced downregulation of Bcl-2 by preventing the decrease of the activated CREB. These results suggest that Ast may improve the survival of cells treated by acetaldehyde via attenuating acetaldehyde-induced inhibition of Akt/CREB pathway.

It has been shown that p38MAPK is activated during 6-hydroxydopamine-induced apoptosis and plays a critical role in promoting apoptotic events in SH-SY5Y cells [44]. Previously, we have reported that acetaldehyde treatment induces p38MAPK activation in a dose dependent manner [27]. Here we observed that Ast treatment significantly reduced the increase of activated p38MAPK induced by acetaldehyde. Similarly, Ast has been found to inhibit the activation of p38MAPK induced by 6-hydroxydopamine or Abeta 25-35 and prevent the loss of cell viability [25,45]. Thus, blocking the activation of p38MAPK may be critical for the neuroprotective effect of Ast. In contrast, it was found that acetaldehyde treatment caused reduction in the levels of activated ERKs, which was largely prevented by Ast pretreatment. As the elevation of activated ERKs has been shown to mediate the inhibition of apoptosis in cultured hippocampal neurons and endothelial cells [46,47], prevention of the loss of activated ERKs may be another mechanism by which Ast inhibits the apoptosis induced by acetaldehyde. It has been reported that the activation of p38MAPK is involved in the suppression of PI3K/Akt signaling pathway and CREB mediated Bcl-2 expression [48,49], while treating cells with ERK inhibitor attenuates the activation of Akt [50,51]. Thus, the inhibition of the activation of p38MAPK and the recovery of ERK activation by Ast treatment may also play a role in modulation of Akt/CREB mediated cell signaling in acetaldehyde-treated cells, eventually leading to the promotion of cell survival.

Oxidative stress is an early event and an important factor in acetaldehyde-induced cytotoxicity and [12,13,14,27]. Ast pre-treatment significantly decreased the production of ROS and MDA while restoring the level of GSH, indicating a significant reduction of oxidative stress in cells treated by acetaldehyde. Therefore, the antioxidant property of Ast may contribute to the protective effect of Ast on acetaldehyde-induced cytotoxicity of SH-SY5Y cells. It is known that ROS stimulates cellular responses involved in both adaptive survival and death including the activation of MAPK and Akt pathways [52,53]. By restoring the redox balance of the cells, Ast may modulate the cell signals in a way that inhibits apoptosis and promotes cell survival.

**Figure 6:**
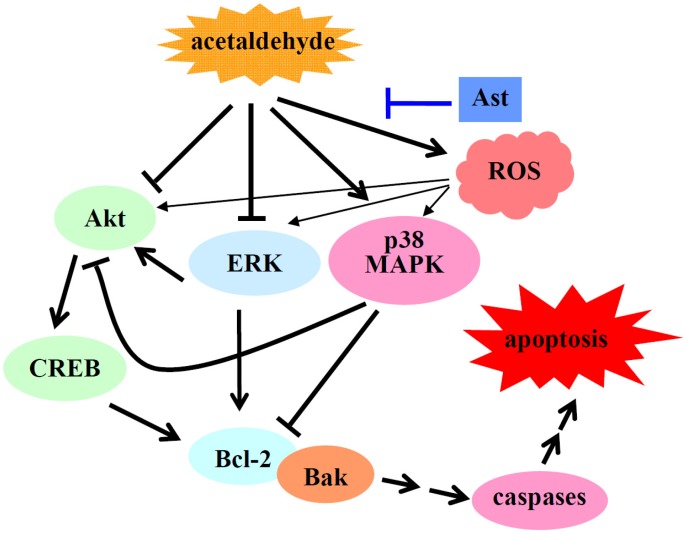
Possible mechanisms by which Ast inhibits acetaldehyde-induced apoptosis and cytotoxicity.

Overall, the results suggest that Ast may inhibit acetaldehyde-induced apoptosis and cytotoxicity by reducing the oxidative stress, preventing the activation of p38 MAPK, and attenuating the inhibition of ERK and Akt/CREB mediated cell signals in acetaldehyde-treated cells (Figure 6).

## 4. Materials and Methods 

### 4.1. Materials

Fetal bovine serum (FBS), streptomycin and penicillin were purchased from Thermo Scientific (Rockford, IL, USA). Dulbecco’s modified eagle’s medium (DMEM) and trypsin were purchased from Invitrogen (Eugene, OR, USA). Ast (3*S*,3′*S*-dihydroxy-β,β-carotene-4,4′-dione) was purchased from Cayman Chemical Company (Ann Arbor, MI, USA). Dimethyl sulfoxide (DMSO) was used as the solvent for Ast. Caspase 3 activity kit, bicinchoninic acid (BCA) protein assay kit, DMSO, BeyoECL plus Western blotting detection system, anti-Akt, donkey anti-goat IgG (H+L) and goat anti-rabbit IgG (H+L) antibodies were purchased from Beyotime Institute of Biotechnology (Haimen, China). MDA assay kit and GSH assay kit were purchased from Nanjing Jiancheng Bioengineering Institute (Nanjing, China). Brandford protein assay kit and anti-phospho-Akt (Ser473) antibody were purchased from Sangon Biotech (Shanghai, China). DCFH-DA was purchased from Sigma Chemical (St. Louis, MO, USA). Antibodies for CREB, phospho-CREB (Ser133), p38MAPK, phospho-p38MAPK (Thr180/Tyr182), p44/p42 MAPK, phospho-p44/p42MAPK (Thr202/Tyr204) were purchased from Cell Signaling Technology (Beverly, MA, USA). Antibody for Bak was purchased from Wanleibio Co., Ltd. (Shanghai, China). Antibody for Bcl-2 was purchased from Boster Institute of Biotechnology (Wuhan, China). Antibody for actin was purchased from Santa Cruz Biotechnology (Dallas, TX, USA).

### 4.2. Cell Viability Assay

Human neuroblastoma SH-SY5Y cells were maintained in DMEM with 10% FBS, 1% streptomycin/penicillin in a CO_2_ incubator at 37 °C, with 5% CO_2_ and 95% air. Cells were seeded in 12-well plates with a density of 4 × 10^4^ per well. The viability of cells was checked by trypan blue exclusion assay.

### 4.3. Hoechst 33258 Nuclear Staining Assay

Apoptotic cells were detected by Hoechst 33258 nuclear staining assay. Cells were fixed for 10 min at room temperature after treatments. The fixed cells were incubated with Hoechst 33258 for 5 min at room temperature and subsequently washed with PBS for three times in the dark. The fluorescence was examined using an Olympus BX53 fluorescence microscope.

### 4.4. Caspase 3 Activity

The activity of caspase 3 was determined using a caspase 3 activity kit according to the manufacturer’s instructions. After treatments, cells were harvested by digesting with trypsin and cell lysates were prepared. To evaluate the activity of caspase 3, cell lysates were incubated with Ac-DEVD-pNA and reaction buffer for 10 h at 37 °C. Substrate cleavage was then measured using a spectrometer at 405 nm.

### 4.5. Measurement of Intracellular Oxidation Stress

Fluorescent probe DCFH-DA was used to determine the changes of the intracellular generation of ROS. After treatments, cells were rinsed three times with PBS, and incubated with 5 μM DCFH-DA at 37 °C for 30 min. The fluorescence was examined using an Olympus BX53 fluorescence microscope. 

For determining the concentrations of MDA and GSH, cells were harvested after the treatments by trypsinization and the total cell lysates were collected. The levels of MDA and GSH in the samples were determined using commercial available kits according to the manufacturer’s instructions. Lipid peroxidation was evaluated by the reaction of MDA with thiobarbituric acid to form a product that was measured at 532 nm using a spectrometer. For assays of GSH, samples were first deproteinized. The levels of GSH were then measured by the reaction of the sulfhydryl group of GSH with 5,5′-dithio-bis-2-nitrobenzoic acid (DTNB) to form a yellow colored product that was measured at 405 nm using a spectrometer. Protein concentrations were determined by the BCA protein assay, and the levels MDA and GSH were normalized by the amount of protein in the total cell lysates. 

### 4.6. Western Blotting Analysis

After treatments, cells were washed twice with PBS and the cell lysates were prepared in cell lysis buffer (Tris 20 mM, NaCl 150 mM, EDTA 1 mM, sodium pyrophosphate 2.5 mM, NaF 20 mM, β-glycerophosphoric acid 1 mM, sodium orthovanadate 1 mM). The supernatants were collected after centrifugation at 14,000× *g* for 10 min at 4 °C and the protein concentration of supernatants was measured. Twenty micrograms of protein extracts were resolved by SDS-polyacrylamide gel electrophoresis then transferred to polyvinylidene difluoride (PVDF) membrane and subsequently incubated with specific primary antibodies. PVDF membrane was washed by Tris buffered saline (TBS) containing 0.1% Tween-20 for three times. For detection, the PVDF membrane was incubated with a horseradish peroxidase-coupled secondary antibody, followed by an enhanced chemiluminescence substrate reaction using BeyoECL plus Western blotting detection system.

### 4.7. Statistical Analysis

All the experiments were carried out in triplicates. Quantitative data are represented as the mean ± SD. Statistical analysis of the data was performed by two-way ANOVA. Least significant difference (LSD) test was used for pairwise comparisons. *p* values of 0.05 were considered statistically significant.

## 5. Conclusions

In summary, Ast treatment is effective in preventing acetaldehyde-induced cytotoxicity in SH-SY5Y cells. In addition, Ast may inhibit acetaldehyde-induced cytotoxicity via inhibition of apoptotic signaling, promotion of cell survival pathways, and suppression of oxidative stress. The study provides evidence that Ast treatment may be beneficial for preventing neurotoxicity associated with acetaldehyde and excessive alcohol consumption.

## Abbreviations

The following abbreviations are used in this manuscript:

CREB	cyclic AMP-responsive element binding protein
MAPK	mitogen-activated protein kinase
ERK	extracellular signal-regulated kinases
ADH	alcohol dehydrogenase
ALDH	aldehyde dehydrogenase
Ast	astaxanthin
ROS	reactive oxygen species
MDA	malondialdehyde
GSH	glutathione
FBS	fetal bovine serum
DMEM	Dulbecco’s modified eagle’s medium
DCFH-DA	2′,7′-dichlorodihydrofluorescin diacetate
PVDF	polyvinylidene difluoride

## References

[1] Harper C. (2009). The neuropathology of alcohol-related brain damage. Alcohol Alcohol..

[2] Langballe E.M., Ask H., Holmen J., Stordal E., Saltvedt I., Selbæk G., Fikseaunet A., Bergh S., Nafstad P., Tambs K. (2015). Alcohol consumption and risk of dementia up to 27 years later in a large, population-based sample: The HUNT study, Norway. Eur. J. Epidemiol..

[3] Harwood D.G., Kalechstein A., Barker W.W., Strauman S., George-Hyslop P.S., Iglesias C., Loewenstein D., Duara R. (2010). The effect of alcohol and tobacco consumption, and apolipoprotein E genotype, on the age of onset in Alzheimer’s disease. Int. J. Geriatr. Psychiatry.

[4] Crabb D.W., Liangpunsakul S. (2007). Acetaldehyde generating enzyme systems: Roles of alcohol dehydrogenase, CYP2E1 and catalase, and speculations on the role of other enzymes and processes. Novartis Found. Symp..

[5] Peng G.S., Yin S.J. (2009). Effect of the allelic variants of aldehyde dehydrogenase ALDH2*2 and alcohol dehydrogenase ADH1B*2 on blood acetaldehyde concentrations. Hum. Genom..

[6] Holownia A., Ledig M., Brszko J.J., Ménez J.F. (1999). Acetaldehyde cytotoxicity in cultured rat astrocytes. Brain Res..

[7] Aberle N.S., Burd L., Zhao B.H., Ren J. (2004). Acetaldehyde-induced cardiac contractile dysfunction may bealleviated by vitamin B1 but not by vitamins B6 or B12. Alcohol Alcohol..

[8] Chaudhry K.K., Samak G., Shukla P.K., Mir H., Gangwar R., Manda B., Isse T., Kawamoto T., Salaspuro M., Kaihovaara P. (2015). ALDH2 deficiency promotes ethanol-induced gut barrier dysfunction and fatty liver in mice. Alcohol. Clin. Exp. Res..

[9] Holownia A., Ledig M., Mapoles J., Ménez J.-F. (1996). Acetaldehyde-induced growth inhibition in cultured rat astroglial cells. Alcohol.

[10] Tokuda K., Izumi Y., Zorumski C.F. (2013). Locally-generated acetaldehyde is involved in ethanol-mediated LTP inhibition in the hippocampus. Neurosci. Lett..

[11] Menegola E., Broccia M.L., Renzo F.D., Giavini E. (2001). Acetaldehyde *in vitro* exposure and apoptosis: A possible mechanism of terato genesis. Alcohol.

[12] Tong M., Longato L., Nguyen Q.G.L., Chen W.C., Spaisman A., Monte S.M. (2011). Acetaldehyde-mediated neurotoxicity: Relevance to fetal alcohol spectrum disorders. Oxid. Med. Cell. Longev..

[13] Jung T.W., Lee J.Y., Shim W.S., Kang E.S., Kim J.S., Ahn C.W., Lee H.C., Cha B.S. (2006). Adiponectin protects human neuroblastoma SH-SY5Y cells against acetaldehyde-induced cytotoxicity. Biochem. Pharmacol..

[14] Clavijo-Cornejo D., Gutiérrez-Carrera M., Palestino-Domínguez M., Dominguez-Pereza M., Nuño N., Souza V., Miranda R., Kershenobich D., Gutiérrez-Ruiz MC., Bucio L. (2014). Acetaldehyde targets superoxide dismutase 2 in liver cancer cells inducing transient enzyme impairment and a rapid transcriptional recovery. Food Chem. Toxicol..

[15] Ahmed F., Li Y., Fanning K., Netzel M., Schenk P.M. (2015). Effect of drying, storage temperature and air exposure on astaxanthin stability from *Haematococcus pluvialis*. Food Res. Int..

[16] Nakajima Y., Inokuchi Y., Shimazawa M., Otsubo K., Ishibashi T., Hara H. (2008). Astaxanthin, a dietary carotenoid, protects retinal cells against oxidative stress *in-vitro* and in mice *in-vivo*. J. Pharm. Pharmacol..

[17] Mcnulty H.P., Byun J., Lockwood S.F., Jacob R.F., Mason R.P. (2007). Differential effects of carotenoids on lipid peroxidation due to membrane interactions: X-ray diffraction analysis. Biochim. Biophys. Acta.

[18] Marin D.P., Bolin A.P., Macedo Rde C., Sampaio S.C., Otton R. (2011). ROS production in neutrophils from alloxan-induced diabetic rats treated *in vivo* with astaxanthin. Int. Immunopharmacol..

[19] Liu X.B., Shibata T., Hisaka S., Osawa T. (2009). Astaxanthin inhibits reactive oxygen species-mediated cellular toxicity in dopaminergic SH-SY5Y cells via mitochondria-targeted protective mechanism. Brain Res..

[20] Chew B.P., Park J.S., Wong M.W., Wong T.S. (1999). A comparison of the anticancer activities of dietary beta-carotene, canthaxanthin and astaxanthin in mice *in vivo*. Anticancer Res..

[21] Pashkow F.J., Watumull D.G., Campbell C.L. (2008). Astaxanthin: A novel potential treatment for oxidative stress and inflammation in cardiovascular disease. Am. J. Cardiol..

[22] Jyonouchi H., Zhang L., Gross M., Tomita Y. (1994). Immunomodulating actions of carotenoids: Enhancement of *in vivo* and *in vitro* antibody production to T-dependent antigens. Nutr. Cancer.

[23] Lu Y.P., Liu S.Y., Sun H., Wu X.M., Li J.J., Zhu L. (2010). Neuroprotective effect of astaxanthin on H_2_O_2_-induced neurotoxicity *in vitro* and on focal cerebral ischemia *in vivo*. Brain Res..

[24] Shen H., Kuo C.C., Chou J., Delvolve A., Jackson S.N., Post J., Woods A.S., Hoffer B.J., Wang Y., Harvey B.K. (2009). Astaxanthin reduces ischemic brain injury in adult rats. FASEB J..

[25] Ikeda Y., Tsuji S., Satoh A., Ishikura M., Shirasawa T., Shimizu T. (2008). Protective effects of astaxanthin on 6-hydroxydopamine-induced apoptosis in human neuroblastoma SH-SY5Y cells. J. Neurochem..

[26] Wen X., Huang A., Hu J., Zhong Z., Liu Y., Li Z., Pan X., Liu Z. (2015). Neuroprotective effect of astaxanthin against glutamate-induced cytotoxicity in HT22 cells: Involvement of the Akt/GSK-3β pathway. Neuroscience.

[27] Yan T.T., Zhao Y., Zhang X. (2016). Acetaldehyde Induces Cytotoxicity of SH-SY5Y cells via inhibition of Akt activation and induction of oxidative stress. Oxid. Med. Cell. Longev..

[28] Gross A., McDonnell J.M., Korsmeyer S.J. (1999). BCL-2 family members and the mitochondria in apoptosis. Genes Dev..

[29] Yang J., Liu X., Bhalla K., Kim C.N., Ibrado A.M., Cai J.Y., Peng T., Jones D.P., Wang X. (1997). Prevention of apoptosis by Bcl-2, release of cytochrome *c* from mitochondria blocked. Science.

[30] Hsu Y.Y., Liu C.M., Tsai H.H., Jong Y.J., Chen I.J., Lo Y.C. (2010). KMUP-1 attenuates serum deprivation-induced neurotoxicity in SH-SY5Y cells: Roles of PKG, PI3K/AKT and Bcl-2/Bax pathways. Toxicology.

[31] Zhang L., Zhao H., Zhang X., Chen L., Zhao X., Bai X., Zhang J. (2013). Nobiletin protects against cerebral ischemia via activating the p-Akt, p-CREB, BDNF and Bcl-2 pathway and ameliorating BBB permeability in rat. Brain Res. Bull..

[32] Esterbauer H., Schaur R.J., Zollner H. (1991). Chemistry and biochemistry of 4-hydroxynonenal, malonaldehyde and related aldehydes. Free Radic. Biol. Med..

[33] Lee R.D., An S.M., Kim S.S., Rhee G.S., Kwack S.J., Seok J.H., Chae S.Y., Park C.H., Choi Y.W., Kim H.S. (2015). Neurotoxic effects of alcohol and acetaldehyde during embryonic development. J. Toxicol. Environ. Health A.

[34] Dewson G., Kluck R.M. (2009). Mechanisms by which Bak and Bax permeabilise mitochondria during apoptosis. J. Cell Sci..

[35] Selzner M., Rudiger H.A., Selzner N., Thomas D.W., Sindram D., Clavien P.A. (2002). Transgenic mice overexpressing human Bcl-2 are resistant to hepatic ischemia and reperfusion. J. Hepatol..

[36] Song X.D., Zhang J.J., Wang M.R., Liu W.B., Gu X.B., Lv C.J. (2011). Astaxanthin induces mitochondria-mediated apoptosis in rat hepatocellular carcinoma CBRH-7919 cells. Biol. Pharm. Bull..

[37] Li J., Dai W., Xia Y., Chen K., Li S., Liu T., Zhang R., Wang J., Lu W., Zhou Y. (2015). Astaxanthin Inhibits Proliferation and Induces Apoptosis of Human Hepatocellular Carcinoma Cells via Inhibition of NF-κB P65 and Wnt/Β-Catenin *in Vitro*. Mar. Drug.

[38] Graupner V., Alexander E., Overkamp T., Rothfuss O., de Laurenzi V., Gillissen B.F., Daniel P.T., Schulze-Osthoff K., Essmann F. (2011). Differential regulation of the proapoptotic multidomain protein Bak by p53 and p73 at the promoter level. Cell Death Differ..

[39] Song X., Wang B., Lin S., Jing L., Mao C., Xu P., Lv C., Liu W., Zuo J. (2014). Astaxanthin inhibits apoptosis in alveolar epithelial cells type II *in vivo* and *in vitro* through the ROS-dependent mitochondrial signalling pathway. J. Cell. Mol. Med..

[40] Kang J.Q., Chong Z.Z., Maiese K. (2003). Critical role for Akt1 in the modulation of apoptotic phosphatidylserine exposure and microglial activation. Mol. Pharmacol..

[41] Socodato R., Brito R., Portugal C.C., de Oliveira N.A., Calaza K.C., Paes-de-Carvalho R. (2014). The nitric oxide-cGKII system relays death and survival signals during embryonic retinal development via AKT-induced CREB1 activation. Cell Death Differ..

[42] Zhou J., Ping F.F., Lv W.T., Feng J.Y., Shang J. (2014). Interleukin-18 directly protects cortical neurons by activating PI3K/AKT/NF-κB/CREB pathways. Cytokine.

[43] Finkbeiner S. (2000). CREB couples neurotrophin signals to survival messages. Neuron.

[44] Gomez-Lazaro M., Galindo1 M.F., Concannon C.G., Segura M.F., Fernandez-Gomez F.J., Llecha N., Comella J.X., Prehn J.H., Jordan J. (2008). 6-Hydroxydopamine activates the mitochondrial apoptosis pathway through p38 MAPK-mediated, p53-independent activation of Bax and PUMA. J. Neurochem..

[45] Wang H.Q., Sun X.B., Xu Y.X., Zhao H., Zhu Q.Y., Zhu C.Q. (2010). Astaxanthin upregulates heme oxygenase-1 expression through ERK1/2 pathway and its protective effect against beta-amyloid-induced cytotoxicity in SH-SY5Y cells. Brain Res..

[46] Pang W., Leng X., Lu H., Yang H., Song N., Tan L., Jiang Y., Guo C. (2013). Depletion of intracellular zinc induces apoptosis of cultured hippocampal neurons through suppression of ERK signaling pathway and activation of caspase-3. Neurosci. Lett..

[47] Yan J., Liu Q., Dou Y., Hsieh Y., Liu Y., Tao R., Zhu D., Lou Y. (2013). Activating glucocorticoid receptor-ERK signaling pathway contributes to ginsenoside Rg1 protection against β-amyloid peptide-induced human endothelial cells apoptosis. J. Ethnopharmacol..

[48] Hui K.Y., Yang Y., Shi K.J., Luo H., Duan J., An J.J., Wu P., Ci Y., Shi L., Xu C. (2014). The p38MAPK-regulated PKD1/CREB/Bcl-2 pathway contributes to selenite-induced colorectal cancer cell apoptosis *in vitro* and *in vivo*. Cancer Lett..

[49] Kim H.J., Oh J.E., Kim S.W., Chun Y.J., Kim M.Y. (2008). Ceramide induces p38MAPK-dependent apoptosis and Bax translocation via inhibition of AKT in HL-60 cells. Cancer Lett..

[50] Wang X., Liu J.Z., Hu J.X., Wu H., Li Y.L., Chen H.L., Bai H., Hai C.X. (2011). ROS-activated p38MAPK/ERK-AKT cascade plays a central role in palmitic acid-stimulated hepatocyte proliferation. Free Radic. Biol. Med..

[51] Guan X.H., Fu Q.C., Shi D., Bu H.L., Song Z.P., Xiong B.R., Shu B., Xiang H.B., Xu B., Manyande A. (2015). Activation of spinal chemokine receptor CXCR3 mediates bone cancer pain through an AKT-ERK crosstalk pathway in rats. Exp. Neurol..

[52] McCubrey J.A., Lahair M.M., Franklin R.A. (2006). Reactive oxygen species-induced activation of the MAP kinase signaling pathways. Antioxid. Redox Signal..

[53] Son Y., Cheong Y.K., Kim N.H., Chung H.T., Kang D.G., Pae H.O. (2011). Mitogen-activated protein kinases and reactive oxygen species: How can ROS activate MAPK pathways?. J. Signal Transduct..

